# Supplementary material for: Acquisition of Immune Privilege in GBM Tumors: Role of Prostaglandins and Bile Salts
Source: Int J Mol Sci. 2023 Feb 6;24(4):3198. doi: 10.3390/ijms24043198 (PMC9958596; doi:10.3390/ijms24043198)
Supplement: Supplementary file 1 [file ijms-24-03198-s001.zip › ijms-2135028-supplementary.pdf]

Supplementary Figure Legends.

Figure S1. Cross-correlation analysis, drawn from three databases, showing the statistical significance of the relationship between PGF_2 synthesis and signalling components. On the left we show that PGF_2 synthesis enzyme, AKR1C3 transcripts are negatively correlated with hypoxia marker ADM and the inhibitor of its receptor, PTGFRN. On the right we show that PGF_2 receptor, PTGFR transcripts are positively correlated with the CXCL12/stem-cell angiogenic pathway. Pearson's correlation shown by number and stars (*) indicate statistical significance with $p<0.05$*, $p<0.01$** and $p<0.001$***.

Figure S2.Cross-correlation analysis, drawing from a pair of databases, showing the statistical significance of the relationship between cell-specific markers, PGH_2 synthetic enzyme transcripts, PTGS1 & PTGS2, with PGD_2 synthetic enzyme transcript HPGDS and PGE_2 synthetic enzyme transcripts. Figure S2A shows that Microglial marker ADORA3 enjoys a much better correlation with PTGS1 and HPGDS, than does neutrophil marker PI3. In contrast, Figure S2A shows that microglial marker ADORA3 enjoys a much poorer correlation with PTGS2 and PTGES, than does neutrophil marker PI3.Pearson's correlation shown by number and stars (*) indicate statistical significance with $p<0.05$*, $p<0.01$** and $p<0.001$***.

Figure S3. Correlation analysis, drawing from a pair of databases, showing the statistical significance of the relationship between components of the complement system and PGH_2 synthetic enzymes and microglial-specific marker ADORA3. Figure S3A shows that Microglial marker ADORA3 transcript levels are highly correlated with complement C3a and C5a receptors, C3AR1 and C5AR1, with complement C3, and with the two enzymes that activate C3 into C3a, CFB and CFD. These latter three components are typically highly expressed in liver. Figure S3B shows that Microglial complement C3a receptor, C3AR1, is far better correlated with enzyme transcripts for PGH_2 synthesis enzyme PTGS1 than with PTGS2. HPGDS, which converts PGH_2 to PGD_2, correlates with C3AR1 and with PTGS2. IRF8 which is elevated by PGD_2 signalling is also highly correlated with C3AR1, PTGS2 and HPGDS. Pearson's correlation shown by number and stars (*) indicate statistical significance with $p<0.05$*, $p<0.01$** and $p<0.001$***.

Figure S4. Cross-correlation analysis, drawing from four databases, showing the superior correlation and statistical significance of microglial-specific marker ADORA3 with PTGS1 and with complement C3a receptor, C3AR1 compared with macrophage marker CD68 with PTGS2 and with complement C5a receptor, C5AR1.
These data suggest that GBM tumors are infiltrated with microg1ia expressing PTGS1, upregulated by activation of C3AR1 by GBM derived C3a. Additionally, tumors are also infiltrated with tumor-associated macrophages expressing PTGS2, upregulated by activation of C5AR1 by GBM derived C5a. Pearson's correlation shown by number and stars (*) indicate statistical significance with $p<0.05$*, $p<0.01$** and $p<0.001$***.

Figure S5. Cross-correlation analysis, drawing from four databases, showing the PGF_2 receptor PTGFR is highly correlated with components of vasculature that have been found to be elevated in the CXCL12/stem-cell angiogenic pathway.
Pearson's correlation shown by number and stars (*) indicate statistical significance with $p<0.05$*, $p<0.01$** and $p<0.001$***.

Figure S6. Cross-correlation analysis, drawing from four databases, showing that sperm-coating heat-shock proteins, HSPA6, DNAJB1 and HSP90B1 are all highly cross-correlated. These three immunosuppressive reproduction-related proteins are highly correlated with neutrophil markers PI3 and CHI3L1, and with PGH_2 synthesis enzyme PTGS2.
Pearson's correlation shown by number and stars (*) indicate statistical significance with $p<0.05$*, $p<0.01$** and $p<0.001$***.

Figure S7. Cross-correlation analysis, drawing from four databases, showing that sperm-coating heat-shock protein, HSPA6, is highly cross-correlated with PGE_2 synthesis enzymes PTGS2 and PTGES, and the down-stream responsive element CEBPD. These transcripts correlated well with neutrophil-specific marker CHI3L1

and TGFβ1, which is down-stream of CEBPD-activation in neutrophils. Pearson's correlation shown by number and stars (*) indicate statistical significance with $p<0.05$*, $p<0.01$** and $p<0.001$***.

Figure S8. Four databases were used to examine the expression of CYP27A1 (Left) and impact of gene transcript levels on GBM patient survival (Right). Levels of CYP27A1 are elevated compared to normal brain tissue in all four datasets. The Kaplan-Meier survival curves, with the rank-order survival statistic, indicate that in three of the four databases the expression of high CYP27A1 produces a statistically significant impact on patient outcome at the $p<0.05$ level. Data and curve generation were generated using the Gliovis platform.

Figure S9. Four databases were used to examine the expression of ACOX2 (Left) and impact of gene transcript levels on GBM patient survival (Right). Levels of ACOX2 are elevated compared to normal brain tissue in all four datasets. The Kaplan-Meier survival curves, with the rank-order survival statistic, indicate that in three of the four databases the expression of high ACOX2 produces a statistically significant impact on patient outcome at the $p<0.05$ level. Data and curve generation were generated using the Gliovis platform.

Figure S10. Cross-correlation analysis, drawing from a pair of databases, showing that IRF4 expression is correlated with the down-stream partner PRDM1 and that these modulators of T-cell function are associated with RORC-Treg markers CTLA4, TNFRSF18 (GITR) and FOXP3. Moreover, IL10 which is released by activated RORC-Tregs also correlates with these five transcripts, Figure S10A. In contrast to IRF4, IRF8 and its down-stream partners SPI1 and TCF4 have a different cell specificity. IRF8, SPI1 and TCF4 correlate with microglial transcripts ADORA3, C3AR1 and CXCR3, Figure S10B. Pearson's correlation shown by number and stars (*) indicate statistical significance with $p<0.05$*, $p<0.01$** and $p<0.001$***.

Supplementary Tables S1-5.
Table S1. %Over/Under representation of PG phenotypes compared with sex-steroids phenotypes
Table S2. %Over/Under representation of PG phenotypes compared with Treg/Hypoxia phenotypes
Table S3. %Over/Under representation of Bile salt phenotype compared with prostaglandin phenotypes
Table S4. %Over/Under representation of Bile salt phenotype compared with Treg/Hypoxia phenotypes
Table S5 %Over/Under representation of Bile salt phenotype compared with sex-steroid phenotypes

Figure S1.

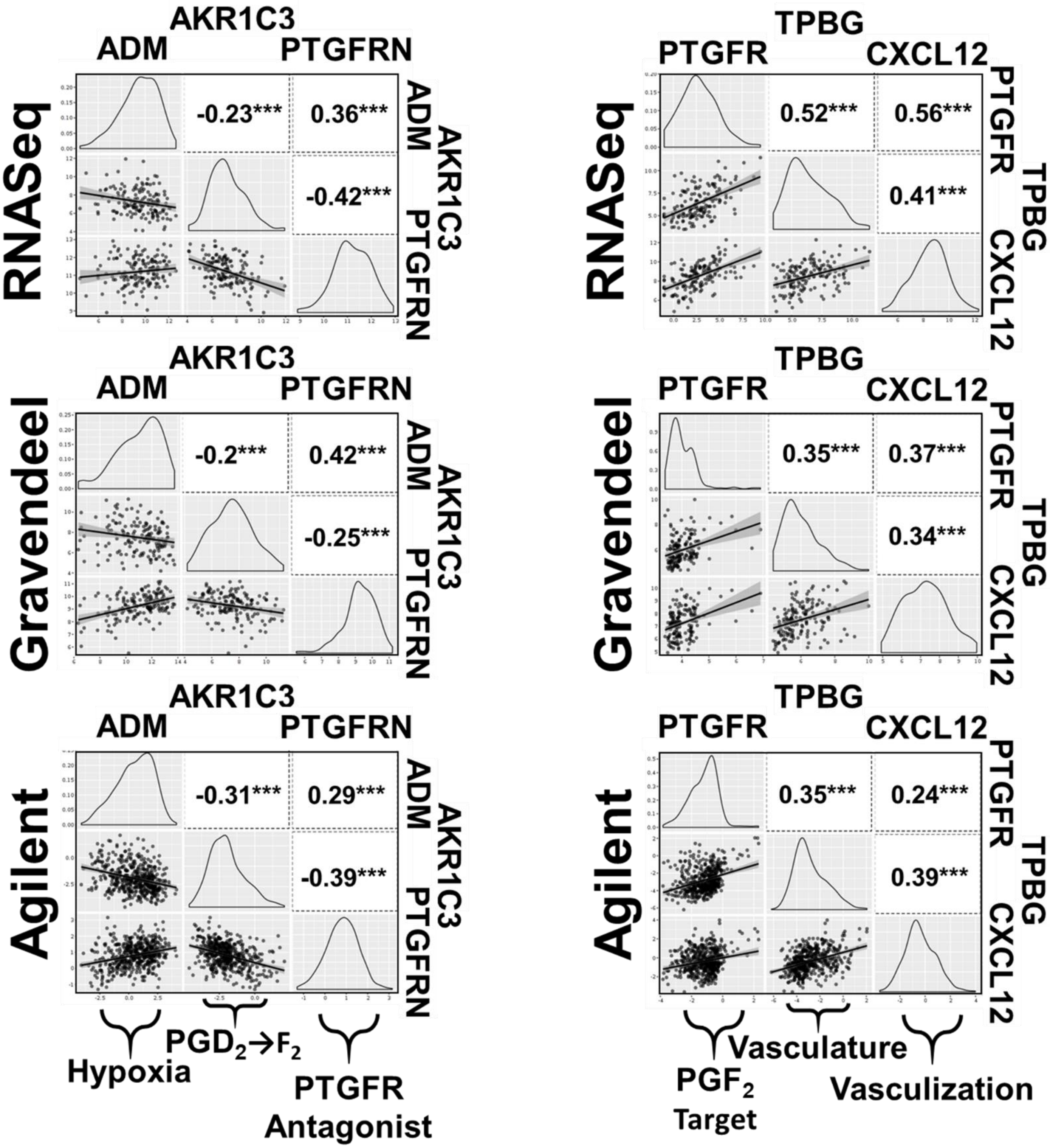
AKR1C3
ADM
PTGFRN
RNASeq
-0.23***
0.36***
-0.42***
Gravendeel
-0.2***
0.42***
-0.25***
Agilent
-0.31***
0.29***
-0.39***
Hypoxia
PGD2→F2
PTGFR Antagonist
TPBG
PTGFR
CXCL12
0.52***
0.56***
0.41***
0.35***
0.37***
0.34***
0.35***
0.24***
0.39***
PGF2 Target
Vasculature
Vasculization

Figure S2.

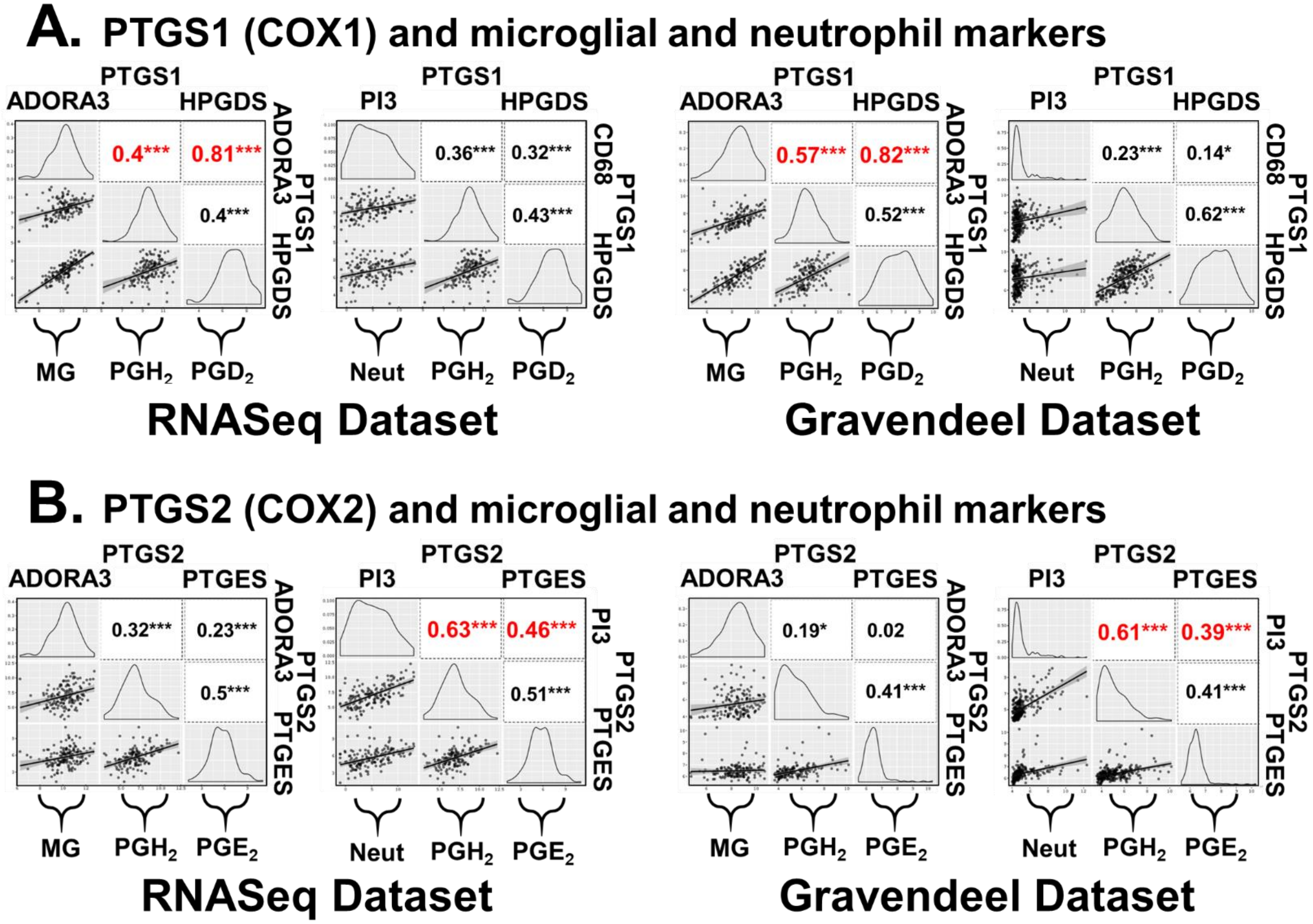
A. PTGS1 (COX1) and microglial and neutrophil markers
PTGS1
ADORA3
HPGDS
0.4***
0.81***
0.4***
PI3
0.36***
0.32***
0.43***
CD68
MG
Neut
PGH$_2$
PGD$_2$
RNASeq Dataset
0.57***
0.82***
0.52***
0.23***
0.14*
0.62***
Gravendeel Dataset
B. PTGS2 (COX2) and microglial and neutrophil markers
PTGS2
PTGES
0.32***
0.23***
0.5***
0.63***
0.46***
0.51***
PGE$_2$
0.19*
0.02
0.41***
0.61***
0.39***
0.41***

Figure S3.

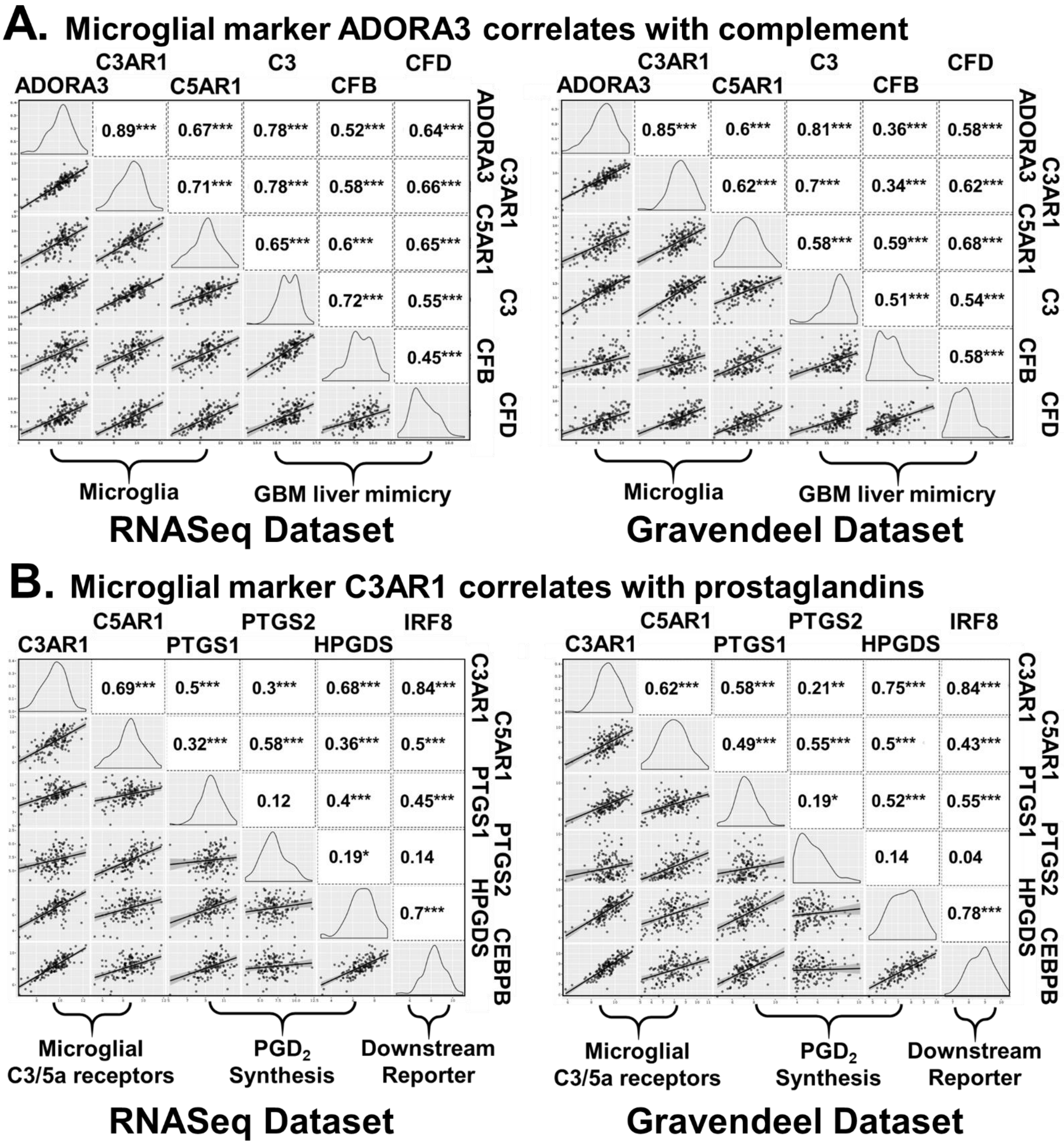
A. Microglial marker ADORA3 correlates with complement
ADORA3
C3AR1
C5AR1
C3
CFB
CFD
Microglia
GBM liver mimicry
RNASeq Dataset
Gravendeel Dataset
B. Microglial marker C3AR1 correlates with prostaglandins
C3AR1
C5AR1
PTGS1
PTGS2
HPGDS
IRF8
CEBPB
Microglial C3/5a receptors
PGD2 Synthesis
Downstream Reporter
RNASeq Dataset
Gravendeel Dataset

Figure S4.

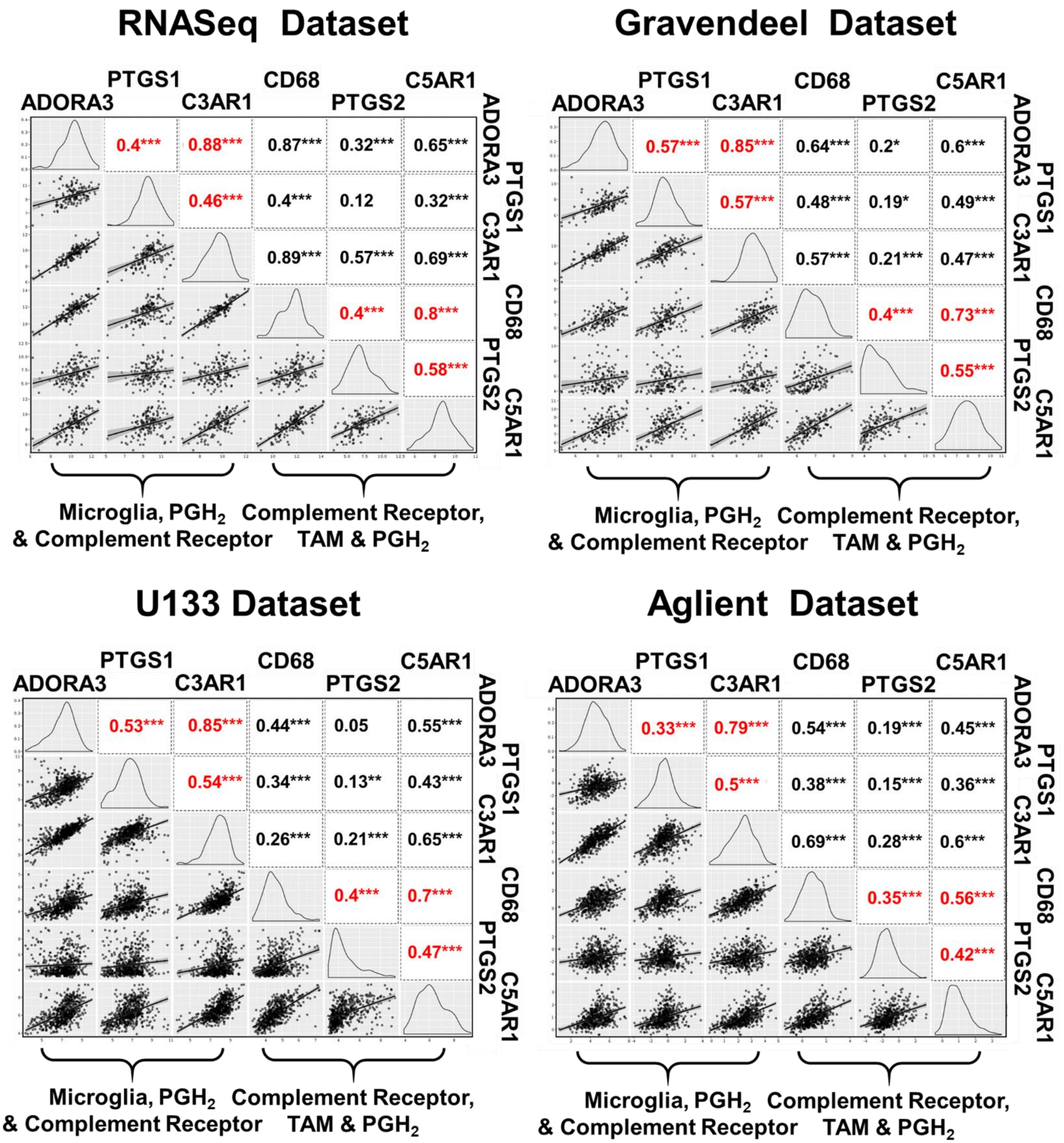
RNASeq Dataset
ADORA3
PTGS1
C3AR1
CD68
PTGS2
C5AR1
0.4***
0.88***
0.87***
0.32***
0.65***
0.46***
0.4***
0.12
0.32***
0.89***
0.57***
0.69***
0.4***
0.8***
0.58***
Microglia, PGH_2
& Complement Receptor
Complement Receptor,
TAM & PGH_2
Gravendeel Dataset
ADORA3
PTGS1
C3AR1
CD68
PTGS2
C5AR1
0.57***
0.85***
0.64***
0.2*
0.6***
0.57***
0.48***
0.19*
0.49***
0.57***
0.21***
0.47***
0.4***
0.73***
0.55***
Microglia, PGH_2
& Complement Receptor
Complement Receptor,
TAM & PGH_2
U133 Dataset
ADORA3
PTGS1
C3AR1
CD68
PTGS2
C5AR1
0.53***
0.85***
0.44***
0.05
0.55***
0.54***
0.34***
0.13**
0.43***
0.26***
0.21***
0.65***
0.4***
0.7***
0.47***
Microglia, PGH_2
& Complement Receptor
Complement Receptor,
TAM & PGH_2
Aglient Dataset
ADORA3
PTGS1
C3AR1
CD68
PTGS2
C5AR1
0.33***
0.79***
0.54***
0.19***
0.45***
0.5***
0.38***
0.15***
0.36***
0.69***
0.28***
0.6***
0.35***
0.56***
0.42***
Microglia, PGH_2
& Complement Receptor
Complement Receptor,
TAM & PGH_2

Figure S5.

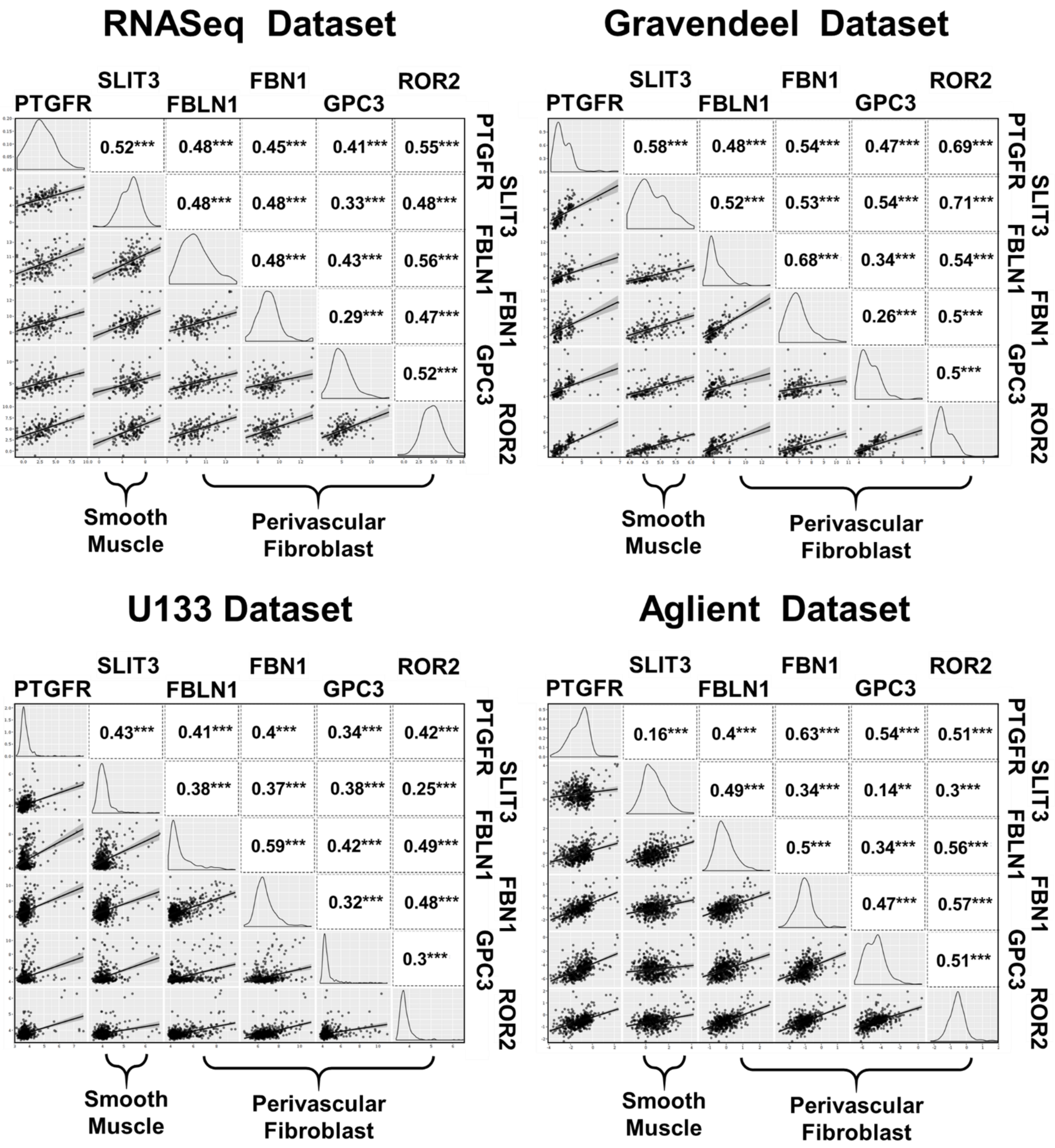
RNASeq Dataset
PTGFR
SLIT3
FBLN1
FBN1
GPC3
ROR2
0.52***
0.48***
0.45***
0.41***
0.55***
0.48***
0.48***
0.33***
0.48***
0.48***
0.43***
0.56***
0.29***
0.47***
0.52***
Smooth Muscle
Perivascular Fibroblast
Gravendeel Dataset
PTGFR
SLIT3
FBLN1
FBN1
GPC3
ROR2
0.58***
0.48***
0.54***
0.47***
0.69***
0.52***
0.53***
0.54***
0.71***
0.68***
0.34***
0.54***
0.26***
0.5***
0.5***
Smooth Muscle
Perivascular Fibroblast
U133 Dataset
PTGFR
SLIT3
FBLN1
FBN1
GPC3
ROR2
0.43***
0.41***
0.4***
0.34***
0.42***
0.38***
0.37***
0.38***
0.25***
0.59***
0.42***
0.49***
0.32***
0.48***
0.3***
Smooth Muscle
Perivascular Fibroblast
Aglient Dataset
PTGFR
SLIT3
FBLN1
FBN1
GPC3
ROR2
0.16***
0.4***
0.63***
0.54***
0.51***
0.49***
0.34***
0.14**
0.3***
0.5***
0.34***
0.56***
0.47***
0.57***
0.51***
Smooth Muscle
Perivascular Fibroblast

Figure S6.

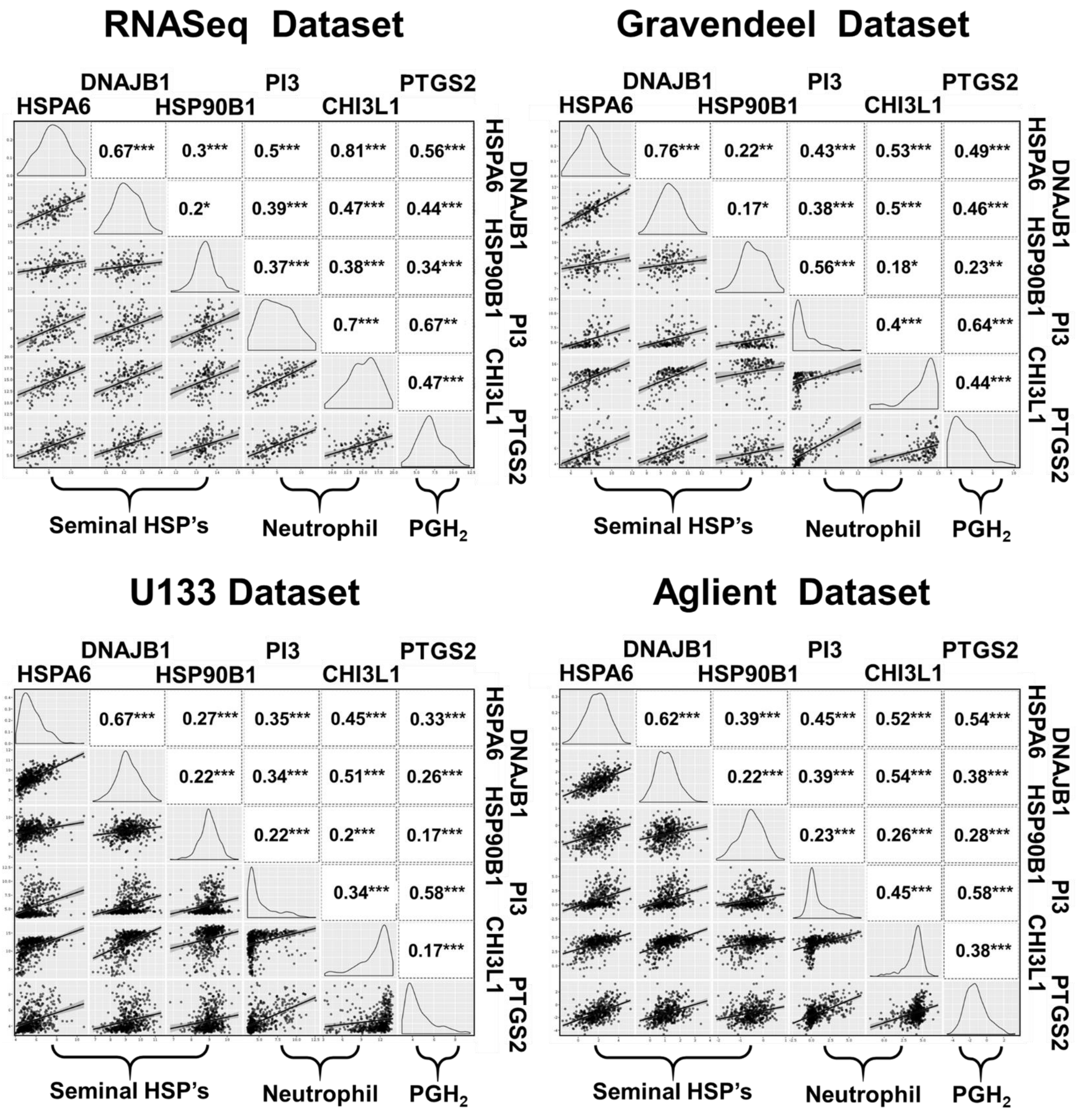
RNASeq Dataset
HSPA6
DNAJB1
HSP90B1
PI3
CHI3L1
PTGS2
0.67***
0.3***
0.5***
0.81***
0.56***
0.2*
0.39***
0.47***
0.44***
0.37***
0.38***
0.34***
0.7***
0.67**
0.47***
Seminal HSP's
Neutrophil
PGH_2
Gravendeel Dataset
HSPA6
DNAJB1
HSP90B1
PI3
CHI3L1
PTGS2
0.76***
0.22**
0.43***
0.53***
0.49***
0.17*
0.38***
0.5***
0.46***
0.56***
0.18*
0.23**
0.4***
0.64***
0.44***
Seminal HSP's
Neutrophil
PGH_2
U133 Dataset
HSPA6
DNAJB1
HSP90B1
PI3
CHI3L1
PTGS2
0.67***
0.27***
0.35***
0.45***
0.33***
0.22***
0.34***
0.51***
0.26***
0.22***
0.2***
0.17***
0.34***
0.58***
0.17***
Seminal HSP's
Neutrophil
PGH_2
Aglient Dataset
HSPA6
DNAJB1
HSP90B1
PI3
CHI3L1
PTGS2
0.62***
0.39***
0.45***
0.52***
0.54***
0.22***
0.39***
0.54***
0.38***
0.23***
0.26***
0.28***
0.45***
0.58***
0.38***
Seminal HSP's
Neutrophil
PGH_2

Figure S7.

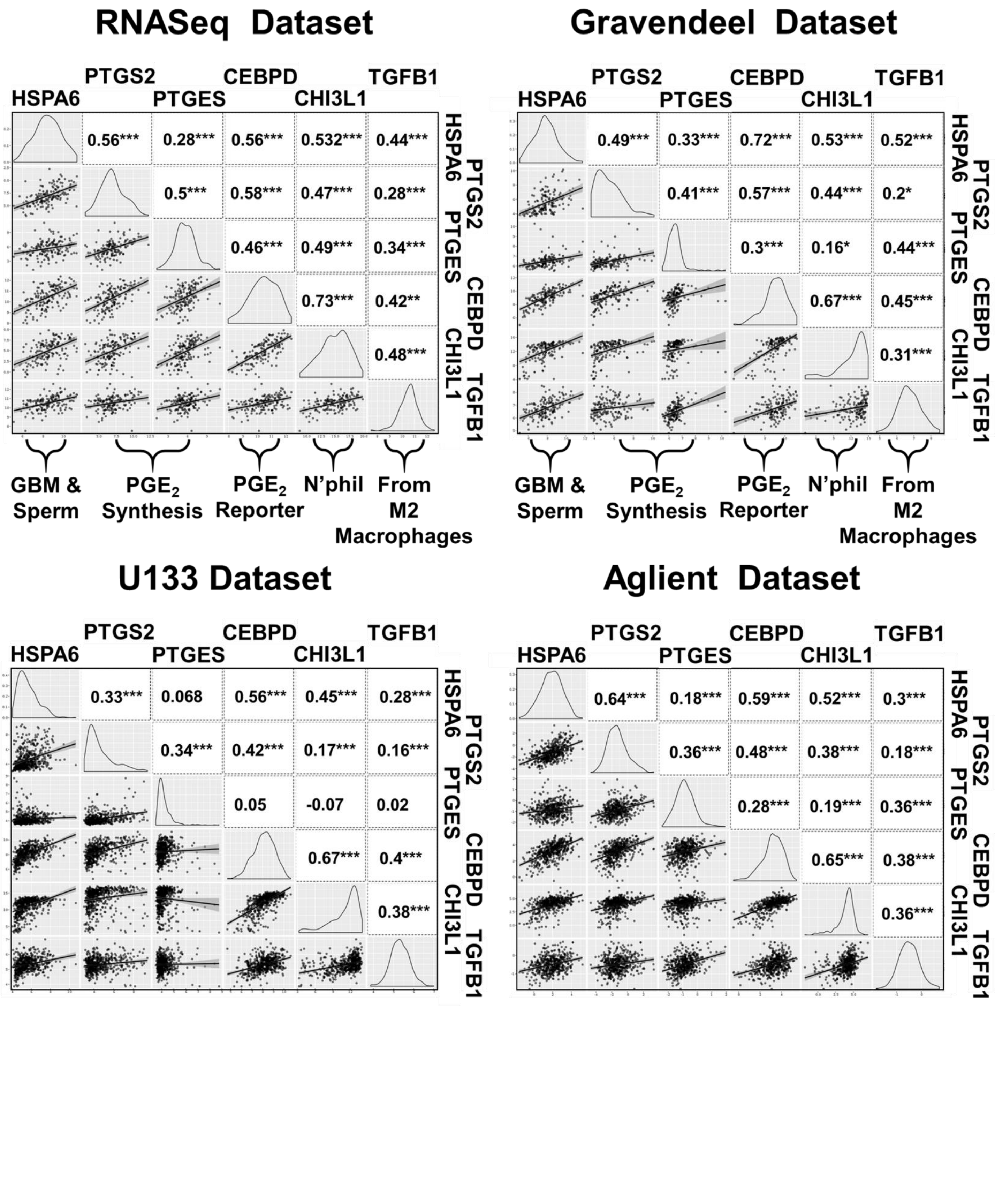
RNASeq Dataset
HSPA6
PTGS2
PTGES
CEBPD
CHI3L1
TGFB1
0.56***
0.28***
0.56***
0.532***
0.44***
0.5***
0.58***
0.47***
0.28***
0.46***
0.49***
0.34***
0.73***
0.42**
0.48***
GBM & Sperm
PGE2 Synthesis
PGE2 Reporter
N'phil
From M2 Macrophages
Gravendeel Dataset
HSPA6
PTGS2
PTGES
CEBPD
CHI3L1
TGFB1
0.49***
0.33***
0.72***
0.53***
0.52***
0.41***
0.57***
0.44***
0.2*
0.3***
0.16*
0.44***
0.67***
0.45***
0.31***
GBM & Sperm
PGE2 Synthesis
PGE2 Reporter
N'phil
From M2 Macrophages
U133 Dataset
HSPA6
PTGS2
PTGES
CEBPD
CHI3L1
TGFB1
0.33***
0.068
0.56***
0.45***
0.28***
0.34***
0.42***
0.17***
0.16***
0.05
-0.07
0.02
0.67***
0.4***
0.38***
Aglient Dataset
HSPA6
PTGS2
PTGES
CEBPD
CHI3L1
TGFB1
0.64***
0.18***
0.59***
0.52***
0.3***
0.36***
0.48***
0.38***
0.18***
0.28***
0.19***
0.36***
0.65***
0.38***
0.36***

Figure S8.

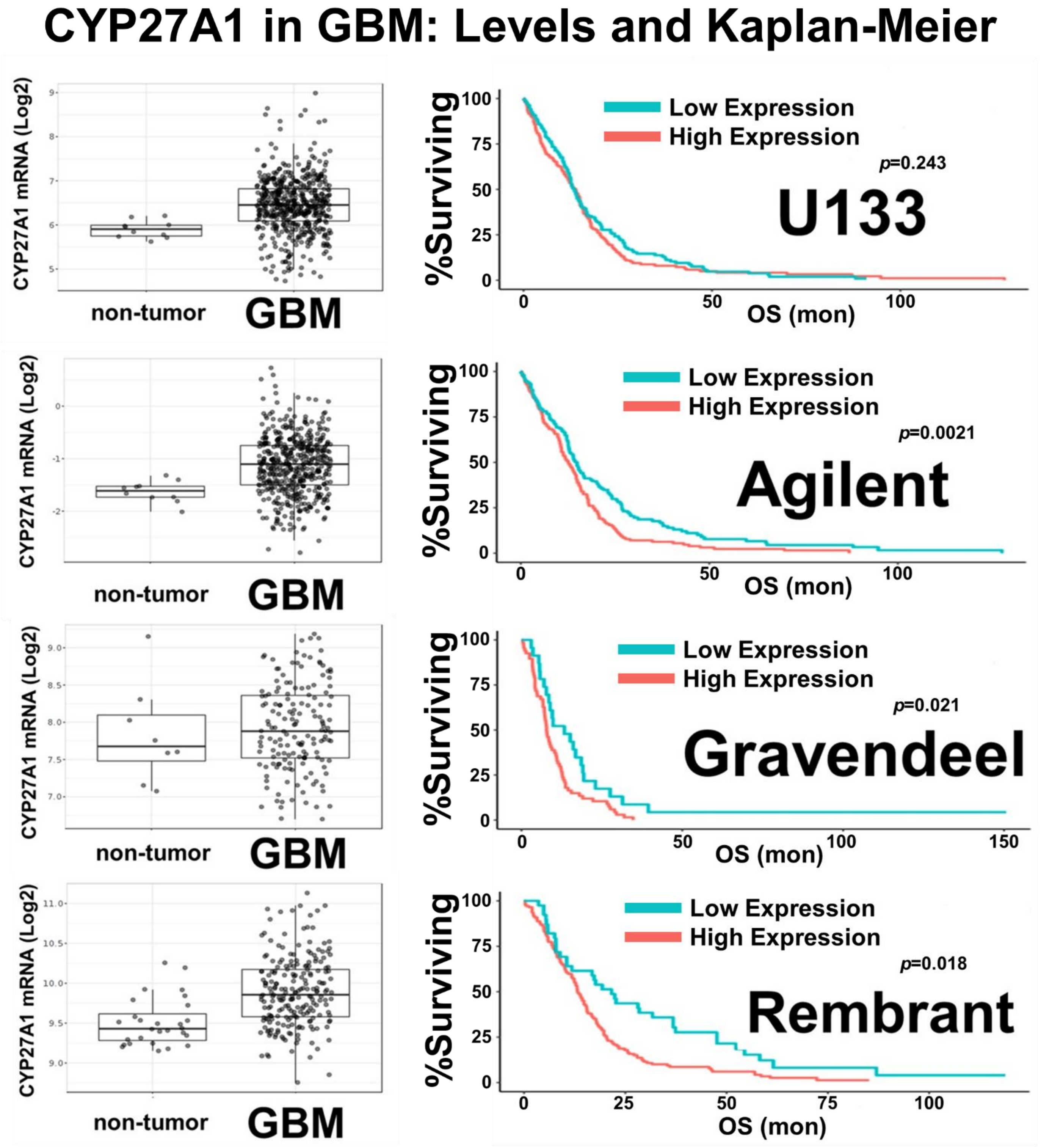
CYP27A1 in GBM: Levels and Kaplan-Meier
CYP27A1 mRNA (Log2)
non-tumor
GBM
%Surviving
OS (mon)
Low Expression
High Expression
p=0.243
U133
p=0.0021
Agilent
p=0.021
Gravendeel
p=0.018
Rembrant

Figure S9.

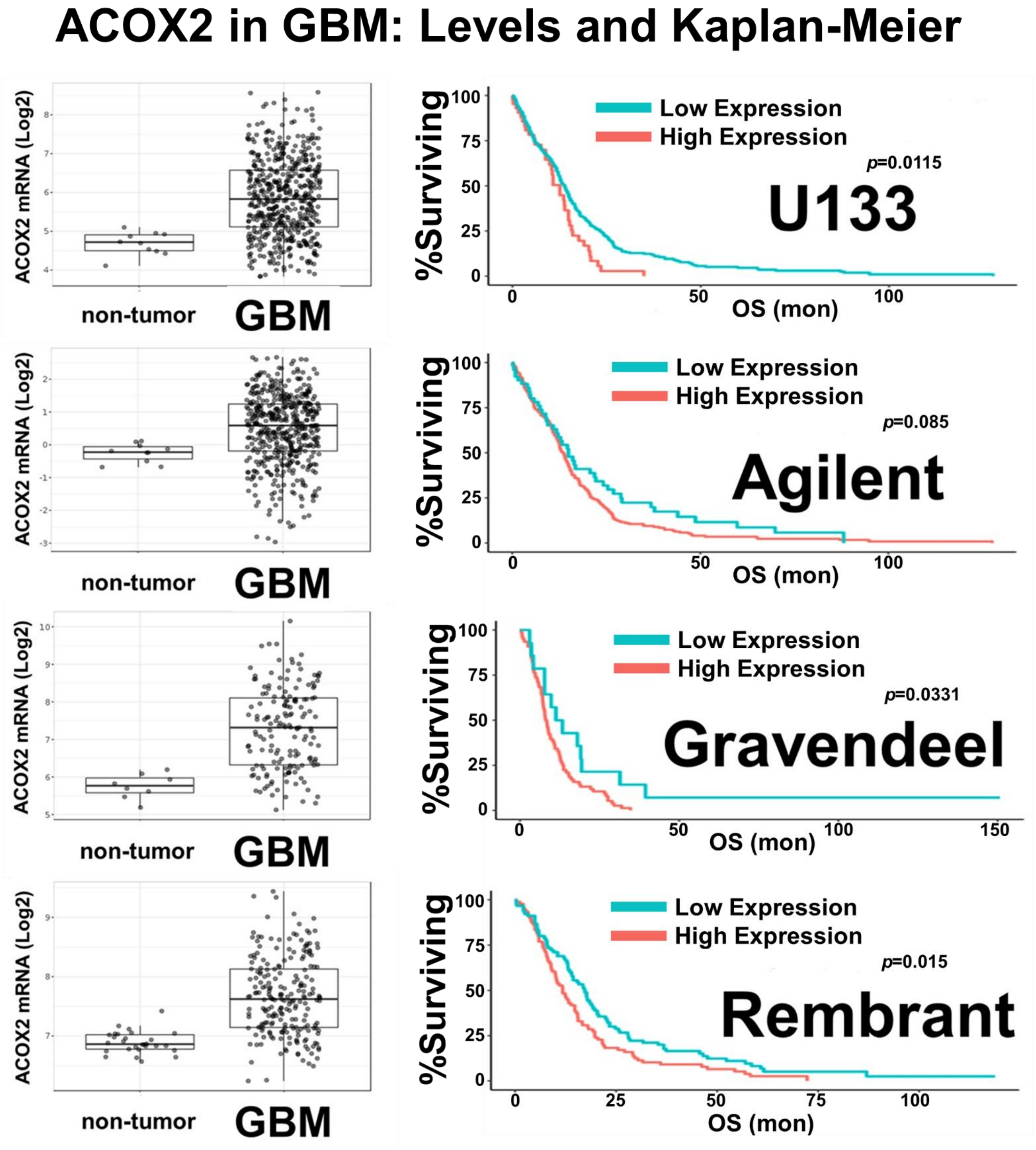
ACOX2 in GBM: Levels and Kaplan-Meier
ACOX2 mRNA (Log2)
non-tumor
GBM
%Surviving
Low Expression
High Expression
p=0.0115
U133
OS (mon)
p=0.085
Agilent
p=0.0331
Gravendeel
p=0.015
Rembrant

Figure S10.

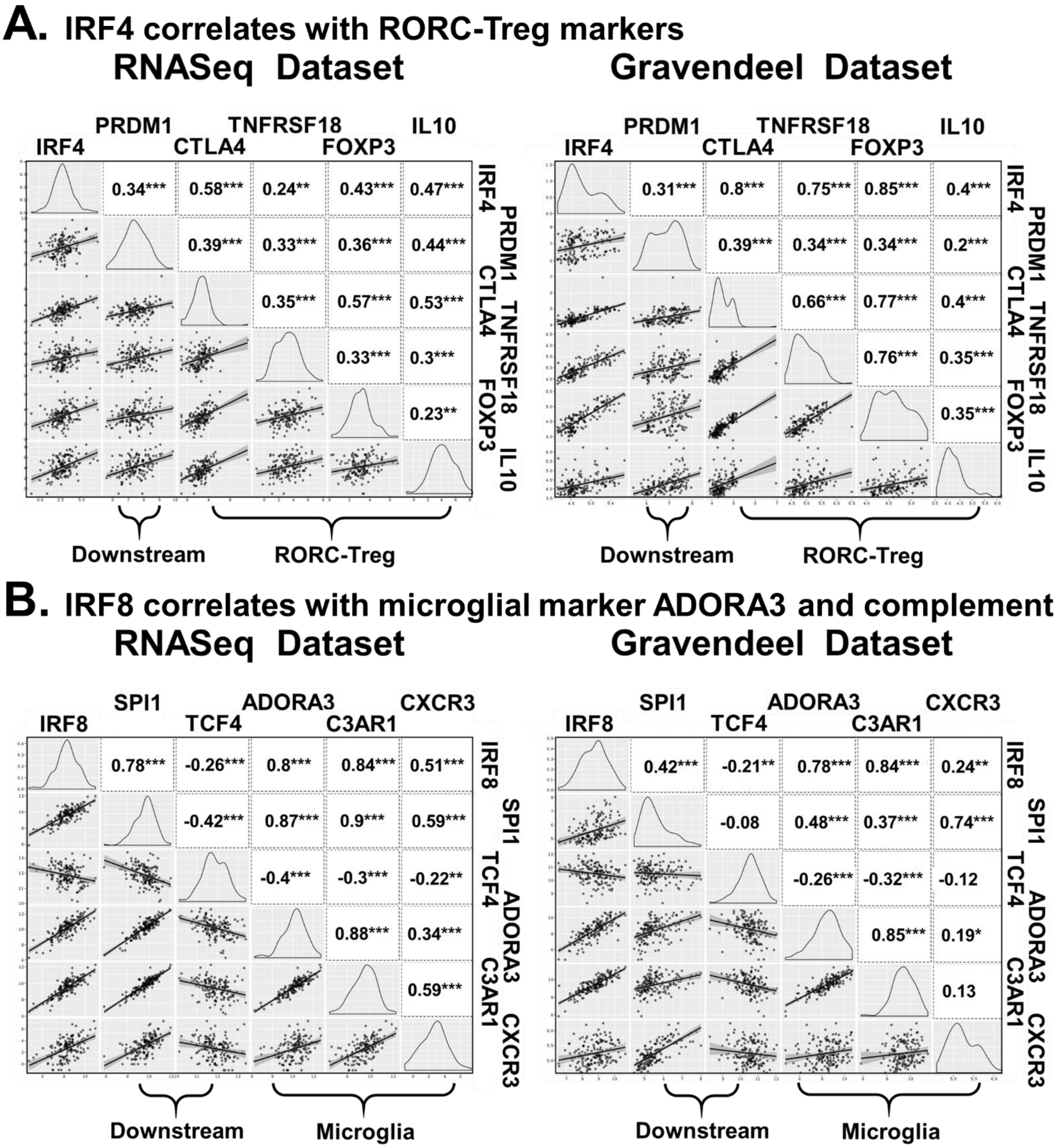
A. IRF4 correlates with RORC-Treg markers
RNASeq Dataset
Gravendeel Dataset
IRF4
PRDM1
CTLA4
TNFRSF18
FOXP3
IL10
0.34***
0.58***
0.24**
0.43***
0.47***
0.39***
0.33***
0.36***
0.44***
0.35***
0.57***
0.53***
0.33***
0.3***
0.23**
0.31***
0.8***
0.75***
0.85***
0.4***
0.39***
0.34***
0.34***
0.2***
0.66***
0.77***
0.4***
0.76***
0.35***
0.35***
Downstream
RORC-Treg
B. IRF8 correlates with microglial marker ADORA3 and complement
RNASeq Dataset
Gravendeel Dataset
IRF8
SPI1
TCF4
ADORA3
C3AR1
CXCR3
0.78***
-0.26***
0.8***
0.84***
0.51***
-0.42***
0.87***
0.9***
0.59***
-0.4***
-0.3***
-0.22**
0.88***
0.34***
0.59***
0.42***
-0.21**
0.78***
0.84***
0.24**
-0.08
0.48***
0.37***
0.74***
-0.26***
-0.32***
-0.12
0.85***
0.19*
0.13
Downstream
Microglia

Supplementary Tables: The Chi Squared *p*=value calculated for row (right) and columns (bottom) are indicated

Table S1. %Over/Under representation of PG phenotypes compared with sex-steroids phenotypes

	% Over/under-representation in subgroup				
Phenotypes	Androgen & Progesterone	Estrogen & Progesterone	Estrogen & Androgen	Asteroidogenic	Row *p*=
PGD_2	128	138	57	70	$4.4*10^{-5}$
PGE_2	117	85	59	137	*0.0014*
PGF_2	70	88	170	78	$1.54*10^{-6}$
Low PG’s	83	87	115	120	*0.24*
Column *p*=	*0.008*	*0.03*	$2.4*10^{-8}$	*0.0038*	All $5.2*10^{-12}$

Table S2. %Over/Under representation of PG phenotypes compared with Treg/Hypoxia phenotypes

	% Over/under-representation in subgroup				
Phenotypes	Treg	Treg&HiF	HiF	Neither	Row *p*=
PGD_2	79	125	125	73	*0.011*
PGE_2	94	102	118	88	*0.51*
PGF_2	106	102	64	125	*0.031*
Low PG’s	125	65	92	116	*0.037*
Column *p*=	*0.174*	*0.051*	*0.018*	*0.05*	0.0003

Table S3. %Over/Under representation of Bile salt phenotype compared with prostaglandin phenotypes

	% Over/under-representation in subgroup				
Phenotypes	PGD_2	PGE_2	PGF_2	Low PG’s	Row *p*=
NR1H4	94	110	105	88	*0.7*
Mixed	103	120	116	52	*0.0066*
GPBAR1	130	79	89	102	*0.053*
No Bile	56	88	85	189	$3.6*10^{-6}$
Column *p*=	*0.011*	*0.156*	*0.43*	$1.25*10^{-6}$	$1.4*10^{-7}$

Table S4. %Over/Under representation of Bile salt phenotype compared with Treg/Hypoxia phenotypes

	% Over/under-representation in subgroup				
Phenotypes	Treg	Treg&HiF	HiF	Neither	Row *p*=
NR1H4	161	137	45	48	$1.184*10^{-11}$
Mixed	68	104	114	117	*0.0499*
GPBAR1	66	95	135	108	*0.00627*
No Bile	107	42	108	143	*0.00085*
Column *p*=	$7*10^{-7}$	*0.0005*	*0.00012*	*0.00012*	All $1.2*10^{-15}$

Table S5. %Over/Under representation of Bile salt phenotype compared with sex-steroid phenotypes

	% Over/under-representation in subgroup				
Phenotypes	Androgen & Progesterone	Estrogen & Progesterone	Estrogen & Androgen	Asteroidogenic	Row *p*=
NR1H4	130	96	122	35	$1.7*10^{-5}$
Mixed	105	92	77	130	0.1
GPBAR1	77	139	106	76	0.012
No Bile	74	68	92	189	$1.06*10^{-5}$
Column *p*=	0.014	0.018	0.17	$3.18*10^{-9}$	All $3.9*10^{-11}$
